# Post-Fire Forest Pulse Recovery: Superiority of Generalized Additive Models (GAM) in Long-Term Landsat Time-Series Analysis

**DOI:** 10.3390/s26020493

**Published:** 2026-01-12

**Authors:** Nima Arij, Shirin Malihi, Abbas Kiani

**Affiliations:** 1 Faculty of Geodesy and Geomatics Engineering, K. N. Toosi University of Technology, Mirdamad St., Tehran 19967-15433, Iran; n.arij@email.kntu.ac.ir; 2 Civil Engineering Department, University of Cambridge, Cambridge CB3 0FA, UK; 3 Faculty of Civil Engineering, Babol Nooshirvani University of Technology, Babol 47148-71167, Iran; a.kiani@nit.ac.ir

**Keywords:** EVI, generalized additive models (GAMs), LOESS, recovery time, Google Earth Engine (GEE), logistic growth model

## Abstract

**Highlights:**

**What are the main findings?**

- Generalized Additive Models (GAMs) consistently outperformed other methods in modeling nonlinear post-fire vegetation recovery across two contrasting ecosystems. This model exhibited the lowest AIC and RMSE values, demonstrating an unparalleled ability to capture multiphase recovery patterns.
- Post-fire recovery trajectories are inherently nonlinear and ecosystem-specific. Australian (MDSF) forests rapidly returned to baseline levels (within ~2 years), whereas California (SMM) forests with a history of recurrent fires failed to fully recover even after 9 years.

**What are the implications of the main findings?**

- This study provides a transferable methodological framework for monitoring forest resilience under intensifying global fire regimes. Integrating Landsat time series with flexible semi-parametric models like GAM offers a powerful approach for recovery assessment.
- Simple linear and logistic models are insufficient for accurately estimating vegetation recovery time, as the findings emphasize the need for adopting more complex methods capable of capturing the heterogeneity of recovery pathways.

**Abstract:**

Wildfires are increasing globally and pose major challenges for assessing post-fire vegetation recovery and ecosystem resilience. We analyzed long-term Landsat time series in two contrasting fire-prone ecosystems in the United States and Australia. Vegetation area was extracted using the Enhanced Vegetation Index (EVI) with Otsu thresholding. Recovery to pre-fire baseline levels was modeled using linear, logistic, locally estimated scatterplot smoothing (LOESS), and generalized additive models (GAM), and their performance was compared using multiple metrics. The results indicated rapid recovery of Australian forests to baseline levels, whereas this was not the case for forests in the United States. Among climatic factors, temperature was the dominant parameter in Australia (Spearman ρ = 0.513, *p* < 10^−8^), while no climatic variable significantly influenced recovery in California. Methodologically, GAM consistently performed best in both regions due to its success in capturing multiphase and heterogeneous recovery patterns, yielding the lowest values of AIC (United States: 142.89; Australia: 46.70) and RMSE_cv (United States: 112.86; Australia: 2.26). Linear and logistic models failed to capture complex recovery dynamics, whereas LOESS was highly sensitive to noise and unstable for long-term prediction. These findings indicate that post-fire recovery is inherently nonlinear and ecosystem-specific and that simple models are insufficient for accurate estimation, with GAM emerging as an appropriate method for assessing vegetation recovery using remote sensing data. This study provides a transferable approach using remote sensing and GAM to monitor forest resilience under accelerating global fire regimes.

## 1. Introduction

Forests are an essential part of terrestrial ecosystems that provide the necessary conditions for the survival of plants and animals as well as the resources needed for human livelihood [1]. Among the benefits of forests, oxygen production, carbon storage, regulation of water and climate, and environmental purification can be mentioned [2]. In recent years, forest disturbances caused by human activities or natural disasters have led to a deforestation [3]. Forest disturbance is primarily caused by overharvesting, forest fires, pest invasions, diseases, and severe weather events [4].

Among these, fire, a natural phenomenon that existed before the emergence of vegetation, has influenced the evolution of ecosystems [5]. Today, forest fires are common in many ecosystems [6], significantly affecting the structure, function, and overall health of forests [6] and the emergence of forest fire patterns results from the complex interactions between vegetation, human activities, and climatic factors [7]. In recent decades, human development and global warming have dramatically increased the frequency and intensity of forest fires [8].

Forest fire damage can have significant effects on human health, the climate, and ecosystems [9]. After a forest fire, one of the key indicators for assessing ecosystem stability and resilience is examining the forest recovery period [10]. Recovery time refers to the period during which a forest ecosystem, after a severe disturbance, returns to its previous state or a condition close to [11]. This concept not only represents the natural ability of the forest to restore its structure and function, but also indicates the degree of forest resilience against recurring environmental stresses [12]. In this regard, unlike short-term data, using time-series data in post-fire monitoring provides the possibility of gradually observing the vegetation reconstruction process [13].

Traditional methods of monitoring and recovery assessment of ecosystems, including field visits, are generally time-consuming, costly, and limited in spatial and temporal coverage, and in many cases cannot be widely used [14]. In contrast, remote sensing technology offers a powerful alternative to field methods for monitoring forest recovery based on the spectral response of vegetation at multiple spatial and temporal scales [15]. The Landsat archive, with more than four decades of observations, has become an efficient tool for monitoring forest disturbances and post-disturbance recovery [16] and can overcome challenges that traditional approaches are unable to address due to logistical and cost limitations [17]. These datasets have suitable temporal and spatial characteristics for analyzing dynamic processes such as recovery and provide an overview of sequential forest changes after a disturbance [18].

The study of the vegetation recovery process after fire has been introduced in recent decades as one of the main focuses of ecological and remote sensing research [13]. Researchers in this field have attempted to identify forest regeneration patterns at different spatial and temporal scales by using time-series data and various analytical methods. A significant part of these studies has focused on evaluating changes in spectral indices and modeling vegetation behavior in the post-fire period, in a way that allows a more accurate estimation of the time required for the ecosystem to return to stable conditions or conditions close to before the disturbance. In this regard, [19] focused on the boreal forests of Siberia to examine post-fire recovery using time-series of NDVI and NDSWIR indices extracted from MODIS data. This study was conducted with the aim of examining the time required for NDVI and NDSWIR signals to return to their pre-fire conditions. Ref. [20] in a study in Grand Hinggan in northeastern China, examined the recovery that occurred in the forest at three levels of completely artificial restoration (full planting), completely natural restoration, and an intermediate state called artificial promotion using Landsat time-series images over a 24-year period. By using spectral indices, especially the Disturbance Index, they identified the occurrence of disturbance (fire) and also tracked the recovery process of the three restoration methods, and found that the recovery rate, the slope of return, and the shape of the trend are more important than the raw values of the indices.

With the development of time-series algorithms, studies became more detailed. Ref. [21] for the first time used non-parametric trend tests such as Mann–Kendall and Theil–Sen slope for analyzing recovery. The important innovation of this approach was examining recovery simultaneously in a qualitative form and a measurable trend slope. They showed that even after a decade, only a portion of the burned areas reach a significant positive trend, and the recovery slope varies greatly between different regions. Following this development, Ref. [22] used the LandTrendr model to extract disturbance and reconstruction segments in time-series. This method for the first time made it possible to segment the recovery trend at the disturbance point, the decline phase, and the return phase. This model showed that forest behavior is more complex than a simple increasing trajectory and may include increases, stops, and even renewed decreases. In fact, recovery is a highly fluctuating curve that can only be analyzed through time-series modeling. Meanwhile, Ref. [23] also showed that recovery can be modeled as a predictable statistical variable. By combining Landsat time-series with machine learning models, they modeled the recovery rate and demonstrated that variables such as precipitation in the second year and temperature in the early post-fire years have a decisive role in changing the trend slope. This study established a link between trend analysis and predicting recovery time.

One of the most important steps in recent literature is the study of [24] in the journal Remote Sensing. This research not only analyzes the recovery trend but, for the first time, uses short-term and long-term statistical recovery metrics to compare two types of disturbances (fire and harvesting). They showed that the type of disturbance plays a fundamental role in the “shape of the return curve,” and these curves can be quantitatively extracted. What makes [24] study remarkable is that the best distinguishing variables between disturbances are trend parameters, not spectral values. In continuation of this research direction, the study of [25] also offers a trend-based approach for analyzing post-fire recovery using long-term Landsat data. This study showed that fire intensity and especially fire recurrence are determining factors in the dynamic behavior of spectral indices, and areas with recurrent fires display unstable and non-stationary trends in NDVI, NDMI, and NBR. By calculating metrics such as Relative Recovery Indicator, the Ratio of %80, and Year-on-Year average, they demonstrated that the recovery capacity of forests ranges from low to moderate, while their vulnerability to future fires remains at a moderate to high level. These results, by emphasizing the importance of trend and the shape of the recovery curve, extend previous approaches and present a more precise picture of the long-term stability of Mediterranean ecosystems after fire.

Recent studies increasingly view post-fire vegetation recovery as a nonlinear and dynamic process that cannot be fully captured by simple trend- or threshold-based approaches. In this regard, Ref. [26] used Sentinel-2 NDVI time series and generalized additive models (GAMs) to investigate vegetation recovery under compound disturbances caused by wildfires and volcanic activity. Their results showed that recovery trajectories are strongly nonlinear and influenced by factors such as distance from the disturbance source and fire history, and that the shape of the recovery curve provides greater ecological insight than absolute NDVI values alone.

At the same time Ref. [27], through trend-based studies, have linked post-fire recovery to climatic drivers such as precipitation, temperature, and fire recurrence by using slope-based metrics and recovery indices. Ref. [28] subsequently investigated the prediction of fire severity using pre-fire time series of remote sensing and meteorological data in France. They found that the Functional Linear Model (FLM), by accounting for the entire temporal trajectory of the variables, achieved higher accuracy in predicting the Relative Burn Ratio (RBR) compared to conventional approaches.

Despite significant advances in analyzing post-fire recovery, the absence of a comprehensive comparative framework for evaluating statistical and time-series methods simultaneously remains one of the fundamental gaps in the research literature. In response to previous studies, each of the methods based on time-series [29], trend analysis [21], spectral indices, and temporal segmentation models such as LandTrendr [25], although providing unique insights into the vegetation recovery process, are not individually capable of explaining all the complex dimensions of post-fire recovery. However, a considerable portion of the research has focused mainly on a single method and has overlooked the systematic comparison of multiple approaches, long-term analyses, and the role of influencing factors such as fire recurrence and trend non-stationarity. Additionally, several studies [26,27,28] have emphasized the importance of modeling recovery trajectories rather than relying solely on comparisons of pre- and post-fire vegetation indices. However, these studies do not explicitly estimate recovery time nor evaluate the robustness of GAM-based results relative to other statistical or time-series approaches. Although such studies successfully demonstrate the climatic control over recovery rates, they often assume quasi-stationary recovery trends and do not integrate GAMs within a comparative framework. Consequently, the relative stability, sensitivity, and accuracy of different statistical and time-series methods for estimating vegetation recovery time particularly in ecosystems affected by recurrent disturbances have not been sufficiently examined.

Therefore, evaluating forest recovery within the context of complex post-fire systems requires using and comparing a spectrum of statistical and time-series approaches that can reveal different aspects of vegetation dynamics.

Based on the identified research gaps and the growing evidence that post-fire vegetation recovery follows nonlinear and time-dependent trajectories, the present study aims to explicitly evaluate how flexible statistical models—particularly generalized additive models (GAMs)—can improve the estimation and interpretation of post-fire recovery dynamics. Specifically, this study seeks to

1. Quantify post-fire vegetation recovery trajectories using long-term Landsat time series and examine how nonlinear, multi-phase recovery patterns differ between ecosystems with contrasting fire regimes.
2. Assess the capability of GAMs to capture nonlinear, time-dependent recovery processes, including rapid early regrowth, mid-term slowdown, and late-stage stabilization, and compare their performance against commonly used approaches such as linear regression, logistic growth models, and LOESS.
3. Evaluate the stability, sensitivity, and robustness of recovery-time estimates derived from GAMs relative to other statistical and time-series methods under different recovery thresholds (95%, 100%, and 105% of baseline conditions).
4. Investigate the role of climatic drivers in shaping recovery trajectories and assess whether GAM-based modeling provides improved insight into climate–vegetation relationships compared to simple correlation or trend-based approaches.

By explicitly integrating GAMs within a comparative modeling framework, this study aims to clarify how methodological choices influence ecological interpretations of post-fire recovery and to identify the most reliable approach for estimating recovery time in fire-affected forest ecosystems.

## 2. Materials and Methods

### 2.1. Study Area

**Figure 1:**
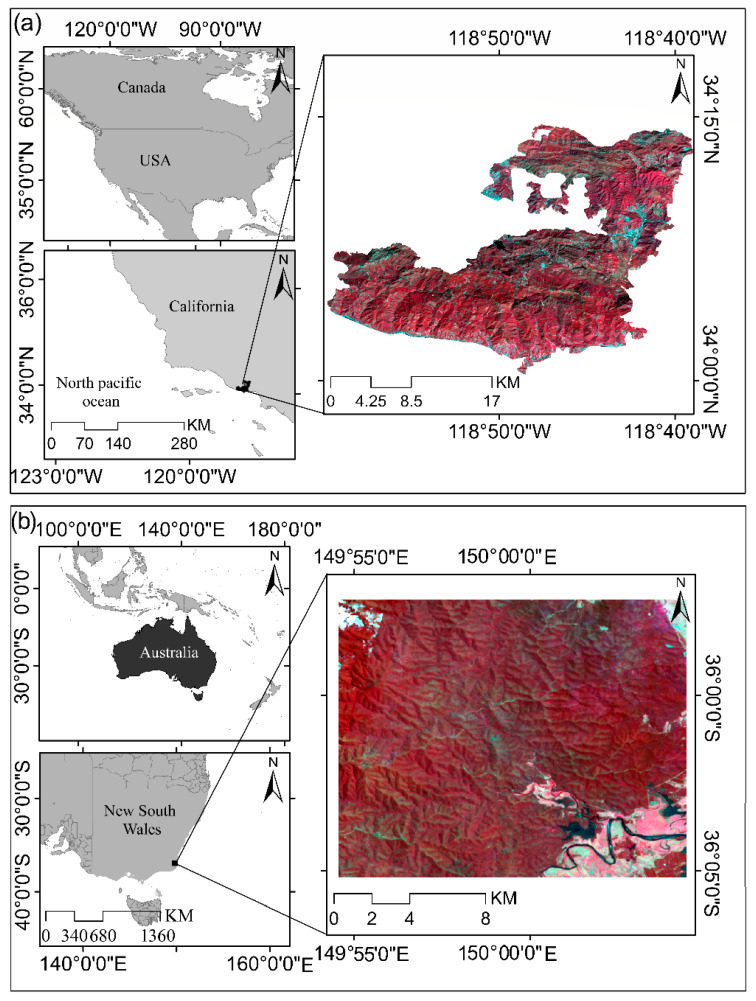
Geographic location and remote sensing view of the two study areas: (**a**) the Woolsey Fire burn area in southern California, United States, located along the Santa Monica Mountains range and the Malibu coastline; (**b**) portions of Moruya State Forest and Dampier State Forest in the state of New South Wales.

We conducted our analysis in two study sites (Figure 1). The first study area includes the Woolsey Fire burn zone in southern California (SoCal), which covers large portions of the Santa Monica Mountains (SMM) range and the coastal areas of Malibu (Figure 1a). This region has a warm Mediterranean climate with an average annual temperature of about 18 °C and an annual precipitation of 350–500 mm, and the topography of the area is diverse, with elevation ranging from 0 to about 860 m above sea level [30,31]. The dominant vegetation consists of chaparral, coastal shrublands, and oak woodlands, which are highly fire-prone in nature [32]. This region has high ecological importance due to its rich biodiversity and the presence of numerous plant and animal species, and parts of it are managed and protected by the National Park Service (NPS) and California state agencies. In addition to its environmental significance, this natural landscape plays an essential role in ecological and social sustainability by providing important ecosystem services such as preventing soil erosion, moderating surface runoff, habitat diversity, and cultural–recreational values [33]. The occurrence of extensive wildfires in 2014 and 2018 caused major changes in the ecosystem structure, vegetation cover, and hydrological dynamics of the area, and these characteristics have made this region one of the most important areas for studies related to fire severity, vegetation regeneration, and the assessment of post-fire environmental impacts. Figure 1, The zoomed-in images of the study areas are prepared based on Landsat satellite data and use a Color Infrared (false color composite) (created using the combination of bands 5: Near-Infrared (NIR), 4: Red, and 3: Green), which displays healthy vegetation in bright red colors, burned areas in darker tones, and non-vegetated areas in cyan to gray colors. This false-color combination enables precise highlighting of structural differences in vegetation cover, detection of burn patterns, and examination of the topography of fire-affected areas.

The second study area is located in parts of the Moruya state forest and Dampier state forest (MDSF) in the state of New South Wales, Australia (Figure 1b). This region is part of the forested landscape of southeastern NSW and, due to the occurrence of extensive wildfires in recent years particularly during the Black Summer Fires (2019–2020) has been severely affected [34]. The region experiences a warm temperate and relatively humid climate, with average annual temperatures ranging between 15 and 17 °C and yearly rainfall typically falling between 900 and 1100 mm. The forested landscape exhibits considerable topographic variation, rising from sea level to elevations exceeding 500 m. Vegetation is dominated by eucalyptus forests (*Eucalyptus* spp.), along with characteristic Australian shrublands and highly fire-prone grasslands communities that collectively shape the behavior and spread of wildfires in the region [35]. Given both the ecological importance of these forests and the extensive damage they have suffered due to fire, this area represents a particularly valuable setting for examining fire severity, monitoring vegetation transformations, and evaluating post-fire ecological impacts within Australia’s forest ecosystems.

### 2.2. Materials

#### 2.2.1. Reference and Satellite Data

We used multitemporal Google Earth Pro images as pseudo-reference data for evaluating the accuracy of our classification due to their high spatial resolution and rich archive, inspired by [36]. We prioritized obtaining reference data in the shortest possible time using available satellite images corresponding to the fire dates. For each region, 100 reference samples were considered as the evaluation dataset. The reference data for MDSF were extracted in April 2020, and for the United States in January 2015 and February 2019.

In the present study, we used the comprehensive Landsat data archive including the TM and OLI sensors available in Google Earth Engine (GEE); an archive that has provided long-term temporal coverage and medium spatial resolution since 1984 [18]. The data used were the Surface Reflectance products in which atmospheric effects have been corrected, and in this regard, only images with less than 60% cloud cover were selected. To effectively reduce cloud-related noise, we applied a median filter according to the approach introduced by [37]. Additionally, to maintain temporal continuity and avoid data scattering, a minimum filter was applied. The median composite was used only in months where multiple semi-cloudy scenes—despite spatial gaps—were collectively sufficient to cover the entire forested region. Although pixel-based compositing methods can retain usable pixels even in cloudier scenes, they often result in spatial inconsistency and image artifacts in heterogeneous landscapes [38]. Therefore, the adopted approach allowed full spatial coverage even when no single scene had adequate quality.

To address the problem of temporal inconsistency, increasing sensor reflectance differences, and to provide standard conversion functions between TM and OLI sensors, the harmonization method recommended by [38] was used, enabling simultaneous and comparable use of Landsat data over time.

#### 2.2.2. Climate Data

In this study, to extract climate variables including precipitation, temperature, and soil moisture, we used the ERA5 Monthly reanalysis dataset belonging to the Copernicus Climate Change Service (C3S) [39], accessible through the Google Earth Engine (GEE) platform. ERA5 is considered a comprehensive collection of atmospheric and surface reanalysis data that, by relying on extensive observational inputs and advanced modeling, provides estimates with high temporal and spatial consistency [40].

The climate data used were extracted on a monthly aggregated basis to allow more accurate analysis of long-term trends and seasonal patterns. These data offer long-term and integrated temporal coverage, suitable spatial consistency for comparison across regions, and a spatial resolution of 0.25 degrees (~30 km) [41], which is appropriate for regional climate analyses. In addition, the capability for direct, automated, and error-free extraction through Google Earth Engine enhanced the quality and uniformity of the data used. All datasets were extracted in a structured and organized manner within the GEE environment. Complete details and descriptive characteristics of the datasets used are reported in Table 1.

### 2.3. Methodology

**Figure 2:**
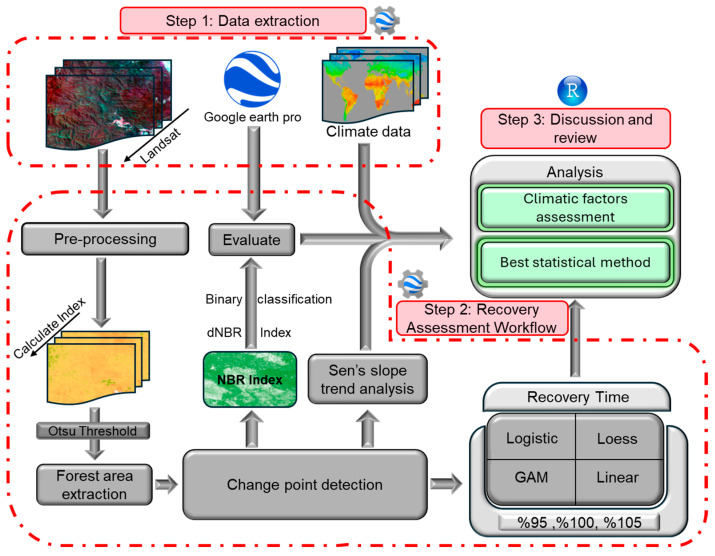
Overall workflow.

Figure 2 illustrates the overall workflow. First, after performing the required preprocessing steps for the Landsat images (including harmonization, cloud filtering, clipping, and median compositing), the spectral indices were calculated. In this study, considering the high vegetation density in the two case studies, the Enhanced Vegetation Index (EVI) was used to extract the vegetation area [42], and after detecting change points using the area obtained from EVI, the Normalized Burn Ratio (NBR) index was applied to generate the burn map, and subsequently, the difference Normalized Burn Ratio (dNBR) map was produced. After calculating dNBR, a Support Vector Machine (SVM) classifier with a Radial Basis Function (RBF) kernel was used to separate burned and unburned pixels. The main parameters of the model, including the error penalty C and the kernel parameter γ, were tuned using Grid Search and k-fold cross-validation to achieve the highest overall classification accuracy [43]. Finally, by applying the SVM model to the dNBR image, a binary map of burned and unburned areas was generated.

To evaluate the accuracy of the resulting map, reference samples collected from high-resolution imagery in Google Earth Pro were used, and metrics such as Overall Accuracy (OA), Kappa, Recall, F1-score, Balanced Accuracy, as well as User’s Accuracy and Producer’s Accuracy were calculated (see [44,45,46,47,48] for more details).

Additionally, for computing the vegetation area using the EVI index, optimal thresholding was performed using the Otsu method [49] combined with an empirical adjustment, as the Otsu thresholding method is sometimes unable to capture region-specific variations. As a result, the combination of Otsu thresholding with this empirical approach ensures more accurate detection of forest vegetation cover under variable conditions [49].

After extracting the area, to examine the pixel-based trend slope according to the study of [14], the Sen’s Slope (SS) method was used to analyze vegetation growth patterns based on burn severity. Subsequently, to assess the role of climatic factors in the rate and intensity of vegetation recovery after fire, climate variables were extracted and resampled monthly to match the spatial scale of the vegetation data. All preprocessing steps—including cloud filtering, harmonization, and median compositing—were performed in Google Earth Engine (GEE).

Additionally, the assessment of recovery time at the 95%, 100%, and 105% return levels is analyzed using different statistical methods. This evaluation is carried out using the criteria Akaike Information Criterion (AIC), Bayesian Information Criterion (BIC), Root Mean Square Error in-sample (RMSE_in), and Root Mean Square Error (cross-validation) (RMSE_CV) in order to select the best model.

### 2.4. Theoretical Background of Methods, Accuracy Assessment & Ecological Metrics

This section presents the theoretical framework and mathematical foundations of the methods used in the recovery modeling analysis, along with the accuracy assessment metrics. The purpose of this section is to provide a clear conceptual basis for understanding how the algorithms operate and the criteria used to evaluate the quality of the results. Statistical methods, by modeling the long-term behavior of vegetation cover and analyzing the patterns of index return, enable a quantitative and comparable estimation of recovery time after fire [50].

#### 2.4.1. Methods

##### Pruned Exact Linear Time (PELT)

The PELT (Pruned Exact Linear Time) method is an exact algorithm for detecting multiple change points, which extracts the optimal time-series segmentation by minimizing a likelihood-based cost function with a linear penalty [51]. Using a pruning rule, this method eliminates non-optimal candidates during computation and reduces computational complexity to linear time, instead of the quadratic complexity of classical methods [52]. PELT, while maintaining full accuracy in finding the global minimum, is highly efficient for long-term time series with multiple changes [53].

##### Linear Model

The linear regression model (Linear) is one of the simplest and most fundamental models for analyzing time-series trends and represents changes in an index as a linear relationship between time and the value of the index [54]. This model follows the general form below:

(1) yt=β0+β1t+εt

where $y_{t}$ is the vegetation index value at time t, $\beta_{0}$ the intercept, $\beta_{1}$ the trend slope (recovery rate), and $\varepsilon_{t}$ the error term with zero mean. This method, due to its high interpretability and direct calculation of the return rate, is suitable when the post-fire index changes are approximately linear, and in such cases, the model computes the time required for the index to reach its pre-fire level using the recursive relationship [54].

##### Logistic

The logistic growth model is one of the most widely used nonlinear models for analyzing the dynamics of vegetation recovery after severe disturbances such as wildfires. The recovery pattern of vegetation in many forests and shrublands naturally follows a three-stage structure consisting of rapid early growth, a decline in growth rate due to increasing biological constraints, and finally reaching a stable or saturated state [55]. The logistic model naturally represents this S-shaped (sigmoidal) pattern and is therefore considered one of the most suitable models for analyzing time-series of spectral indices such as NDVI, EVI, and NBR after fire [56].

The general form of the logistic model for describing changes in a vegetation index $y\left(t\right)$ over time is expressed as:

(2) $$y\left(t\right)=\frac{K}{1+e^{-r\left(t-t_{0}\right)}}$$

In this equation, K is the upper limit or ecological carrying capacity, representing the stable post-recovery value of the vegetation index; r is the growth rate or recovery speed—higher values indicate faster ecosystem return; $t_{0}$ is the inflection point, representing the time when vegetation regrowth reaches its maximum rate; and e is the Napierian constant [57]. These parameters enable quantification of the recovery process, with each reflecting a specific aspect of ecosystem regeneration. To estimate the recovery time at 95%, 100%, and 105% levels, the inverse form of the logistic model can be used. By substituting the target value y into the equation below, the return time is obtained:

(3) $$t=t_{0}-\frac{1}{r}\ln\left(\frac{K}{y}-1\right)$$

##### Locally Estimated Scatterplot Smoothing (LOESS)

The LOESS method is a non-parametric smoothing technique based on local regression that extracts the main trend of time-series without needing to assume any specific functional form. In this method, for each time point, a low-degree polynomial is fitted based on neighborhood data with distance-based weighting [58].

This technique is suitable for satellite-based datasets that typically contain noise, seasonal fluctuations, and abrupt changes, and it is capable of revealing real signals by removing random oscillations [58]. The key parameter of this method is the span, which determines the degree of smoothing, and its proper selection plays a crucial role in accuracy [14]. The main advantage of LOESS is its high flexibility and its ability to represent complex recovery patterns. However, due to its non-parametric nature, it does not directly provide extractable ecological parameters [14].

##### Generalized Additive Model (GAM)

The GAM model is a flexible statistical approach for modeling nonlinear relationships in time-series data, capable of representing complex [35] and multi-stage vegetation trajectories without assuming a predefined functional form [59]. In this method, time is modeled using a smoothing function:

(4) $$y\left(t\right)=\beta_{0}+f\left(t\right)+\varepsilon_{t}$$

where f(t) is a smoothing function estimated from the data using splines. This capability allows GAM to accurately model sudden changes after fire, seasonal fluctuations, temporary pauses in recovery, and long-term trends simultaneously. Compared to linear models, GAM performs much better when dealing with non-stationary behavior, variable slopes, and nonlinear vegetation return. Additionally, its ability to extract trend components and identify inflection points or recovery phase transitions is one of its key strengths [60].

### 2.5. Accuracy Assessment & Ecological Metrics

To select the most robust and accurate model for estimating vegetation recovery time, a set of evaluation metrics including AIC, BIC, RMSE-in, and RMSE-CV was used. Each of these metrics assesses a different aspect of model performance, goodness of fit, and generalizability, which are summarized in Table 2. In addition, several ecological indicators were employed to quantify ecosystem response traits such as recovery speed, resilience, and disturbance impact, providing a complementary perspective on post-fire vegetation dynamics.

## 3. Results

### 3.1. Disturbance and Burn Severity Patterns

**Figure 3:**
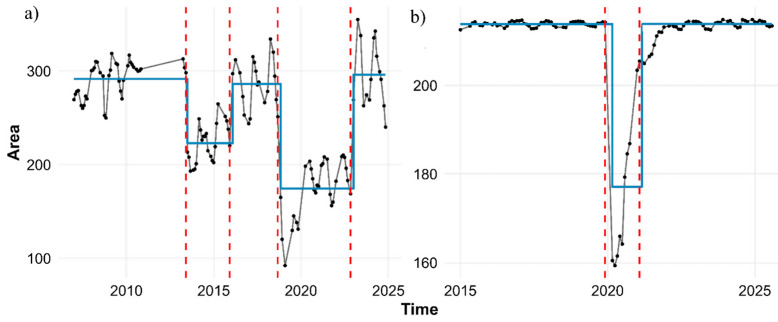
Results of change-point detection using the PELT algorithm with MBIC penalty for the two forest regions: (**a**) SMM and (**b**) MDSF, using area extracted from the EVI index. In both panels, the black line represents the original time-series data, and the step-wise blue lines (piecewise constant) represent the estimated mean levels in each detected segment. The vertical red dashed lines indicate the change points identified by the MBIC criterion.

Long-term changes in forest dynamics were quantified using statistical approaches. The forest area for the MDSF region was obtained from 2015 to 2024, and for the SMM region from 2007 to 2024 (for region-specific results, the reader is referred to Appendix A). After measuring the area, we used the PELT method to accurately identify change points (Figure 3). To evaluate the performance of different models in detecting change points, three criteria—AIC, BIC, and MBIC—were used.

According to Figure 3, two sharp decreases in forest area occurred in the SMM in 2014 and 2019, and in the MDSF region a significant decrease occurred in 2019. The outputs showed that the AIC- and BIC-based models produced identical structures, including five segments for SMM forests and three segments for MDSF, whereas the MBIC criterion identified a more suitable number of segments aligned with official reports. Moreover, analysis of the penalty value in the PELT algorithm showed that as the penalty (β) increases, the number of detected change points decreases and the model tends toward simpler structures. Among these, MBIC which uses a stronger penalty achieved the most appropriate balance between the number of change points and segment stability (Appendix B). Accordingly, MBIC showed the best performance in detecting change points.

**Figure 4:**
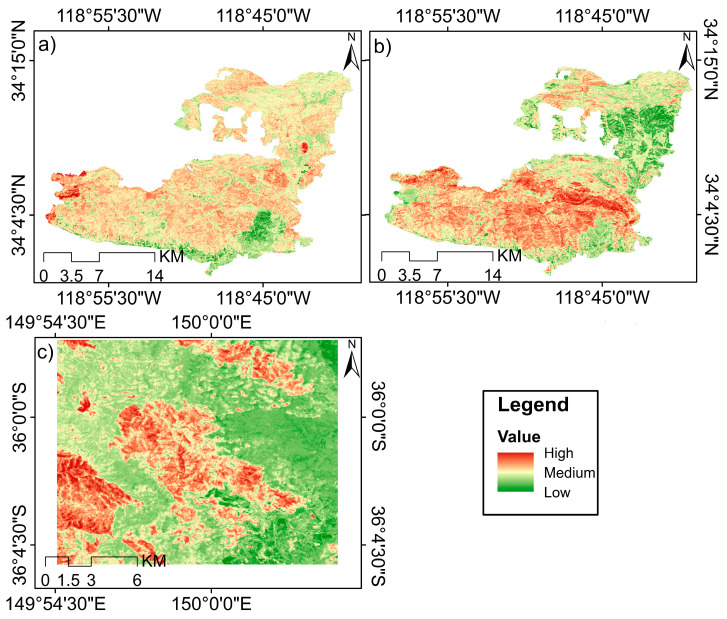
Burn-severity maps derived from the DNBR index for major wildfire events in the SMM and MDSF. Panel (**a**) shows DNBR-based burn severity for the 2014 wildfire in SMM; (**b**) the burn-severity map for the 2018 wildfire in the same region; and (**c**) the burn severity associated with the 2019 wildfire in the MDSF. In all panels, areas with high DNBR values (red) indicate severe burn severity, while medium (yellow) and low (green) values indicate moderate and low severity, respectively.

After detecting change points using the PELT method, the DNBR index was used to analyze fire severity in the affected areas, because it enables spatial assessment of burn severity (Figure 4). To evaluate the accuracy of burn-severity maps derived from DNBR, the data were validated using a binary classification approach based on the SVM algorithm. The evaluation results showed that the SVM model performed satisfactorily in distinguishing burned and unburned areas, with Kappa coefficients of 0.90 for MDSF and 0.87 for the SMM at the two-time steps. These values indicate high agreement between the DNBR-derived maps and reference data and ensure sufficient accuracy for subsequent spatial analyses (more information in Appendix B).

After verifying the accuracy of the DNBR maps, the spatial patterns of burn severity were examined. According to Figure 3a, burn severity in this event was heterogeneous and showed considerable spatial variability. The central and southwestern parts of the region exhibit high DNBR values, indicating severe burning and widespread vegetation loss. In contrast, the eastern and northern portions fall mostly within low-to-moderate severity classes, indicating limited fire impact in these areas. In Figure 3b, the extent and continuity of burn severity show a marked increase compared to 2014. High and continuous DNBR values in the eastern, northeastern, and central parts of the region indicate that the 2018 wildfire not only affected a larger area but also spread with more uniform intensity throughout the region. The greater prevalence of moderate-to-high severity classes compared to 2014 reflects stronger structural damage to vegetation cover in that year. Meanwhile, Figure 3 shows an entirely different pattern from the two SMM wildfires, with a notable spatial continuity of areas exhibiting severe burn severity. This distribution indicates highly aggressive fire behavior and a high level of resulting damage.

After examining spatial burn-severity patterns and their distribution using the DNBR index, the next step involved using the Sen’s Slope method to quantify the trend of forest-cover changes over the study period.

**Figure 5:**
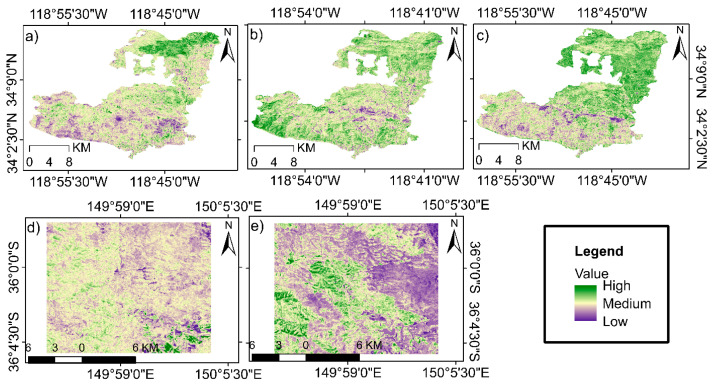
Sen’s slope maps derived from the EVI index, showing pixel-based vegetation-dynamics trends in the study regions. Panels (**a**–**c**) show the spatial distribution of Sen’s slope values for the SMM corresponding to the periods 2007–2014, 2014–2018, and 2018–2024, respectively. Panels (**d**,**e**) show Sen’s slope estimates for the MDSF at the periods 2015–2019 and 2019–2024. Higher slopes (green) indicate positive vegetation trends and improvement; moderate slopes indicate moderate changes; and lower slopes (purple) indicate declining vegetation trends.

Sen’s Slope results showed that the studied regions experienced distinct temporal behaviors following wildfire events. In the SMM, the trend prior to the 2014 wildfire (Figure 5a) exhibited significant positive changes across most areas, especially in the north, while the southern part showed either stable or negative trends. After the 2014 wildfire, the Sen slope indicated a steady increase, with the positive slope clearly larger than before 2014 (Figure 5b). Finally, after the extensive 2018 wildfire, the trend reorganized again. Analyses show that in the northern and central parts, the rate of vegetation change remained positive and stable, while in the southern parts, the changes generally displayed very slight positive slopes with minimal fluctuations (Figure 5c).

In the MDSF, Sen’s Slope analysis revealed a temporal pattern distinct from the SMM regions. The pre-fire slope (Figure 5d) was mostly stable, with slight decreases in some areas, indicating minimal vegetation change in the pre-disturbance period. However, after the 2019 wildfire, the fire-affected areas displayed significant positive slopes (Figure 5e), with the increased Sen slope indicating the onset and intensification of forest-cover regeneration and regrowth. These patterns demonstrate that the intensity and extent of wildfires directly influence the rate and uniformity of vegetation recovery, affecting the temporal dynamics of each part of the region individually.

To examine the role of climatic variables in long-term vegetation changes, the correlations between time-series of temperature, precipitation, and soil moisture with the EVI index were calculated for the SMM and MDSF. The results showed substantial differences between the two regions (Table 3). In the SMM, none of the climatic variables exhibited significant correlation with vegetation changes. Pearson and Spearman correlation values for temperature were −0.045 and −0.088, respectively—both statistically insignificant (*p* > 0.3). Precipitation also showed no identifiable dependency pattern with vegetation cover, with correlation values near zero (*p* > 0.76). For soil moisture, although correlation coefficients were slightly higher and positive (r = 0.07–0.13), these values were also insignificant and did not indicate a determining role in vegetation dynamics.

In contrast, in the MDSF, temperature showed the strongest correlation with vegetation changes; the Spearman coefficient (r = 0.51, *p* < 10^−8^) indicates a highly significant and positive relationship. Precipitation, similar to the SMM, showed no significant correlation (*p* > 0.27). For soil moisture, although both Pearson and Spearman coefficients were negative, the lack of statistical significance (*p* > 0.11) prevents drawing a definitive conclusion about its role.

### 3.2. Post-Fire Recovery Modeling

To evaluate changes in forest cover and the recovery process following disturbance, the time-series data from the SMM and MDSF regions were modeled across different time periods. Four modeling approaches—linear regression, GAM, the logistic growth model, and LOESS—were applied to the data. In this analysis, three threshold levels were also examine, 95% of the baseline, the baseline (100%), and 1.05× the baseline, in order to assess recovery dynamics under different intensity levels (For more information about the choice of the LOESS span and the selection of k in GAM, refer to Appendix B).

For the SMM region, all models consistently identified the drop point on 15 June 2013. This simultaneity indicates that the shock to the time series was abrupt and severe, and that the data structure was sufficiently coherent across all models to reflect the same response (Appendix B). After this period, the values entered a long-term phase of decline and fluctuation at a level considerably lower than the baseline—a period that lasted for almost nine years.

**Figure 6:**
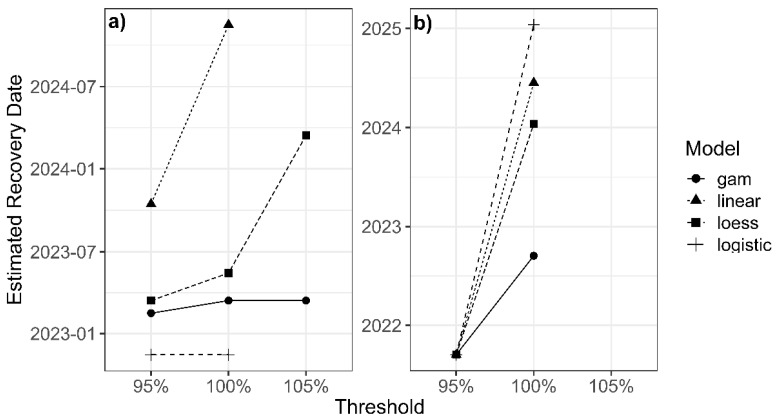
Estimated recovery dates at three recovery thresholds (95%, 100%, 105%) for four statistical models (linear, LOESS, logistic, and GAM for (**a**) the SMM and (**b**) estimated recovery dates for the MDSF. The plots illustrate how model choice and threshold level influence the projected recovery timeline.

Analysis of the recovery point for the 95% baseline threshold (approximately 285.1) showed (Figure 6a) that all four models recorded the start of the recovery phase on 15 November 2022 a point that can be considered the beginning of the structural restoration of the time series. However, the actual time of reaching the threshold differs among the models. The Logistic model detects the threshold crossing simultaneously with the start of recovery, i.e., in November 2022. In contrast, the LOESS and GAM models record threshold attainment in March 2023 and February 2023, respectively. The Linear model, with a more conservative behavior, estimates a later time: October 2023.

**Figure 7:**
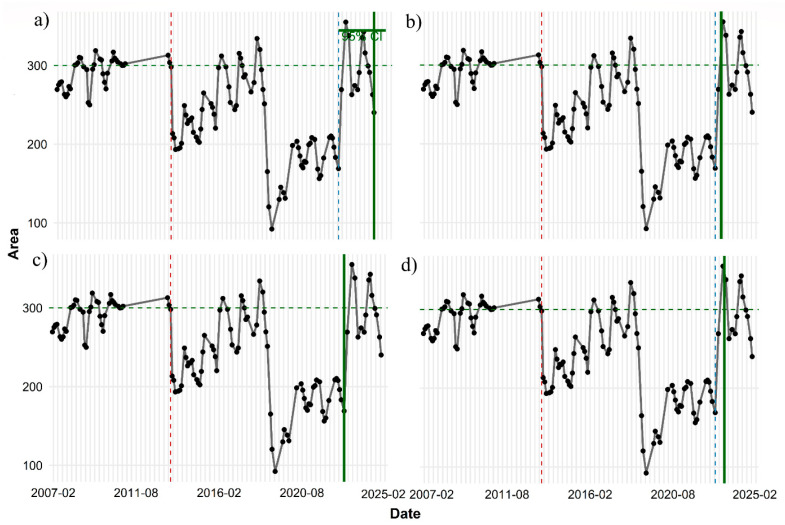
Observed time series of area values and estimated recovery dates under the 100% recovery threshold using four statistical approaches (**a**) Linear regression, (**b**) GAM, (**c**) Logistic growth model, and (**d**) LOESS. In each panel, black points and the grey connecting line represent observed values, the green dashed horizontal line indicates the estimated baseline (300.1), and the red dashed vertical line marks the disturbance date. The solid green vertical line shows the model-specific estimated recovery date.

Recovery relative to the 100% baseline threshold (equal to 300.1) was assessed next (Figure 7). At this level, the date 15 November 2022 is again identified as the start of the upward trend; however, reaching the baseline shows much greater variation among models. The Logistic model reports the same November 2022 date as the point of baseline crossing, indicating a sharp rebound after the decline. In contrast, the GAM model estimates baseline recovery in March 2023, and LOESS in May 2023, whereas the Linear model provides a much later date—November 2024—indicating a far more gradual and delayed recovery prediction.

Finally, examination of the 1.05× baseline threshold (approximately 315.1) shows that attainment of this level occurs only in the LOESS and GAM models, while the Linear and Logistic models record no crossing of this threshold. GAM suggests an earlier date (March 2023) and LOESS a later one (March 2024) (Figure 6a; see Appendix B).

Time-series analysis for the MDSF region shows that the decline-and-recovery pattern exhibits orderly and coherent behavior, and the models fully agree in identifying the key change points. According to the results, the drop point was identified by all four models on exactly the same date: 15 December 2019. This simultaneity indicates that the shock to the system was sudden and substantial, and was consistently reflected in the data structure. After this decline, the time series shifted to a lower level than the baseline and remained there for some time (Figure 6b).

Analysis of the 95% threshold shows that the threshold level—approximately 203.2—was recovered on a single shared date for all models: 15 September 2021 (Figure 6b). This consistency across all models indicates that the MDSF region returned to 95% of its pre-disturbance condition approximately 21 months after the initial drop. Thus, early recovery was rapid and stable, and the models reported no significant differences in the timing of reaching this level.

**Figure 8:**
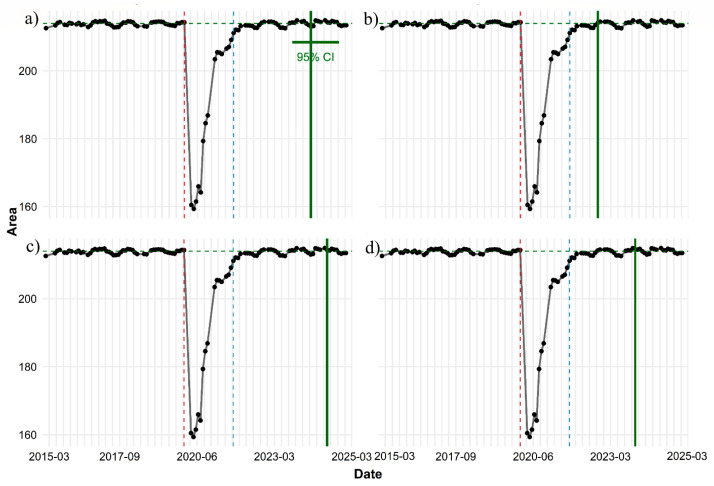
Observed time series of area values and estimated recovery dates under the 100% recovery threshold for MDSF, using four statistical approaches: (**a**) Linear regression, (**b**) GAM, (**c**) Logistic growth model, and (**d**) LOESS. In each panel, the black points and grey connecting line depict the observed time series, the green dashed horizontal line represents the estimated baseline (213.96), and the red dashed vertical line marks the disturbance date. The solid green vertical line indicates the model-specific estimated recovery date, with the blue dashed vertical line showing the estimated trough date prior to recovery.

In the assessment of full baseline recovery, although all models identify the same recovery onset (15 September 2021), the actual date of reaching the baseline differs among them (Figure 8). The GAM model reports the fastest recovery, identifying 15 September 2022 as the baseline return date. The LOESS model estimates 15 January 2024, the Linear model 15 June 2024, and the Logistic model a later date: 15 January 2025. This variation indicates that although the general recovery pattern is similar across models, differences in sensitivity to data fluctuations lead to a relatively wide range of baseline-recovery.

At the 105% threshold of the baseline value (approximately 224.6), the results are fully consistent across models: none of the models show that the MDSF region reached this level (Figure 6b). To complete the temporal recovery analysis, a set of quantitative recovery metrics was also extracted for the SMM and MDSF regions in order to characterize the numerical features of post-fire vegetation regrowth (Table 4).

The quantitative recovery metrics for the SMM and MDSF regions provide a complementary view of post-disturbance dynamics and clarify the numerical characteristics of the regeneration process at the inter-regional scale. In both regions, the date of minimum vegetation cover was consistent across models: 15 February 2019 in the SMM and 15 April 2020 in MDSF. Additionally, the time to half recovery was identical across models for each region—14 months for the SMM and 6 months for MDSF —indicating differences in initial regrowth speed between the two ecosystems.

The range of recovery rates in the SMM was estimated to be approximately 1.70 to 4.99, and in the MDSF region approximately 0.96 to 3.04. This variation among models reflects a spectrum of regeneration patterns, ranging from moderate recovery to faster return trajectories. The ecological resistance index in the SMM ranged from 0.36 to 1.22, and in the MDSF region from 0.94 to 1.01, while the hysteresis index was reported between 0.112 and 0.147 in the SMM and between 0.53 and 0.55 in the MDSF region. The vegetation value at the time of recovery was also about 169 to 346 in the SMM and about 211 to 214 units in the MDSF region.

Finally, to evaluate the performance of the four modeling approaches used in analyzing vegetation trends, a set of standard metrics was examined (Table 5). These metrics allow comparison of model fit quality, structural complexity, and generalizability between the SMM and MDSF regions.

The results indicate that in the SMM region, the GAM model produced the lowest AIC and BIC values among the four models and also had the smallest in-sample RMSE (RMSE_in = 20.39); therefore, in terms of fit and structural complexity, it performs better than the other models. The LOESS model also shows acceptable performance with a relatively low RMSE (24.93) and an AIC lower than those of the Linear and Logistic models. In contrast, the Logistic model has the highest prediction error (RMSE_in = 47.2) and the largest AIC value, indicating weaker fit in the SMM trends. Although the Linear model has a simpler structure, it still produces higher in-sample error compared to GAM and LOESS. The RMSE_cv pattern also corroborates these findings and highlights the differences in out-of-sample performance among the models.

In the MDSF region, the difference in model performance is more pronounced. The GAM model shows by far the best performance among the four models, recording the lowest AIC (46.69) and the lowest in-sample error (RMSE_in = 0.328). This performance, combined with relatively low RMSE_cv values, indicates the strong capability of GAM in representing vegetation trends while maintaining good generalizability. The LOESS model also performs well and ranks second. In contrast, the Logistic model in the MDSF region presents the highest AIC and RMSE_in values, and similar to the SMM results, records the weakest model performance. The Linear model results also show that although this model has a simpler structure, it ranks lower in predictive accuracy compared to GAM and LOESS.

## 4. Discussion

This study examined the dynamics of post-fire vegetation recovery in two fire-affected forest regions in the SMM and MDSF, using long-term Landsat time series, statistical analyses and nonparametric trends, and a set of recovery-modeling frameworks. By integrating spectral indices, change-point detection, ecological metrics, and climatic correlations, the findings provide new insights into the temporal complexity of recovery trajectories and the differential sensitivity of ecological systems to fire severity and climatic variability. By comparing the performance of four modeling approaches—Linear, Logistic, LOESS, and GAM—this study also offers a rare methodological assessment in the context of post-fire recovery research. While previous studies have often focused on a single method or a single spectral index [22,50], this work introduces recovery modeling as a multidimensional problem that benefits from comparative analysis. Therefore, the results of this study extend the existing literature on post-disturbance forest dynamics and highlight gaps regarding the impact of model choice on ecological interpretations. To strengthen the interpretation of recovery trajectories, our framework explicitly models post-fire recovery as a nonlinear and multi-phase process. By using GAM, the shape of the recovery pathway is learned directly from the data rather than assumed a priori, enabling smooth transitions in growth rate, multiple inflection points, and the identification of distinct recovery phases. This allows us to characterize the full recovery trajectory, rather than solely estimating a single time-to-recovery metric.

### 4.1. Ecological Insights into Disturbance and Recovery

The disturbance patterns observed based on PELT confirm that abrupt and large changes in vegetation cover are well aligned with known fire events (Figure 3), which is consistent with previous findings that accurate statistical segmentation algorithms outperform visual or threshold-based methods in detecting post-fire transitions [52,69]. We found that the MBIC penalty produced the most ecologically meaningful number of change points, in line with [51,70], who showed that stronger penalties reduce over-segmentation and lead to more stable disturbance maps. In the SMM site, two major declines in 2014 and 2018 indicated severe disturbances corresponding to the Woolsey fire history, whereas the MDSF site showed a single, clear major change point associated with the Black Summer fires of 2019–2020. These observations are consistent with global studies showing that Mediterranean-type ecosystems (such as southern California) often experience frequent, high-frequency fires that reshape vegetation structure multiple times [70], whereas moist sclerophyll forests in MDSF typically have longer fire-return intervals but more intense events [71].

The burn-severity patterns derived from DNBR maps (Figure 4) further refine the ecological significance of these disturbances. The spatial heterogeneity of the SMM fires, especially in 2018 (Figure 4b), reflects the known patch-scale heterogeneity in chaparral–mixed oak systems, where fuel discontinuity and topographic complexity generate highly variable fire intensities [30]. In contrast, the MDSF site exhibited more continuous, high-severity patches (Figure 4c), consistent with [72], who showed that eucalyptus forests burned during the Black Summer fires experienced more uniform crown consumption due to prolonged pre-fire drought. Drought-stressed trees, even those that are generally fire-adapted like eucalyptus, showed increased mortality and more uniform canopy loss during these extreme fires

Furthermore, spatial analysis of burn severity based on dNBR and its subsequent validation using SVM classification revealed contrasting implications for recovery trajectories in the two regions. Greater heterogeneity and fragmented high-severity patches in the 2014 SMM fire created a mosaic of recovery conditions in which areas with lower burn severity can act as regeneration nuclei through seed dispersal and vegetative expansion from unburned refugia [73]. In contrast, the extensive and high-severity burns of the 2019 Australian fire, characterized by large, contiguous areas with high dNBR values, likely posed greater challenges to recovery by depleting seed sources and causing widespread mortality of resprouting organs.

Trend analysis using Sen’s slope revealed clear contrasts between pre- and post-fire vegetation trajectories (Figure 5). According to Figure 5a–c, vegetation signals in the northern sectors were increasing, while southern areas experienced stagnation or decline. This heterogeneity reflects the land-use mosaic in the Santa Monica Mountains, where microclimate, species composition, and fragmentation patterns influence local growth rates [33]. After the 2014 and 2018 fires, however, recovery patterns shifted toward more stabilized positive trends, indicating strong regenerative capacity in chaparral systems—consistent with resprouting and obligate seeding strategies in these ecosystems [30]. In contrast, the MDSF site showed no clear trend before the fire (Figure 5d), but slopes became strongly positive after 2019, reflecting rapid canopy repair and refoliation of eucalyptus species [74]. The presence of moderate negative slopes in surrounding areas, however, indicates local constraints such as soil burn effects or hydrological disruption, consistent with [75], who noted that post-fire soil moisture deficits can prolong canopy thinning.

Climatic correlations also revealed system-specific sensitivities (Table 3). At the SMM, none of the climatic variables—temperature, precipitation, or soil moisture—showed statistically significant correlations with EVI dynamics. This pattern points to a system in which post-fire recovery is mainly constrained by non-climatic factors. In Mediterranean-type ecosystems such as southern California chaparral, post-fire regeneration is often dominated by seed-dependent shrubs and resprouting species whose early recovery depends more on seed banks, root reserves, and fire-adapted life histories than on short-term climatic fluctuations [30]. The relatively high recovery rates and resilience indices calculated for the SMM (Table 4) further support the idea that the native flora is well adapted to a high-frequency fire regime. We found that short-term post-fire recovery in Mediterranean forests, at least in the early years, is not directly constrained by drought, and biotic factors play the predominant role

By contrast, at the MDSF, temperature showed a strong positive correlation with vegetation recovery (Spearman ρ = 0.513, *p* < 0.001). This pattern is consistent with the ecology of eucalyptus-dominated forests, where higher temperatures can stimulate vigorous epicormic regrowth from trunks and branches, a characteristic trait of these ecosystems [76]. The shorter time to half recovery observed at this site (6 months vs. 14 months in the SMM, Table 4) also highlights the high regrowth capacity of Australian forests. However, this temperature dependence also implies greater vulnerability to future climate change. Increasing heat stress and vapor pressure deficit may eventually shift this positive relationship toward a negative one, threatening system resilience. The lack of significant correlation with precipitation and soil moisture, though initially surprising, is likely related to the relatively humid climate of the region or to the coarse spatial resolution of ERA5, which may not capture fine-scale soil moisture heterogeneity [77].

Moreover, as shown in Table 4 and Figure 6, recovery thresholds provide additional insight into resilience dynamics. Both systems reached the 95% threshold faster than the 100% threshold, as expected, but the contrast between them is notable. The SMM took nearly nine years to return to its pre-fire baseline, whereas the MDSF site recovered much more rapidly. We found that systems with obligate resprouters, such as eucalyptus forests, rebuild vegetation structure much faster than seed-dependent shrublands, consistent with [78]. None of the models reached the 105% threshold in the MDSF (Figure 6b), indicating that despite rapid recovery, the system did not surpass the baseline.

Ecological metrics also place these findings within a broader context. Higher hysteresis values at the MDSF site (Table 4) indicate stronger path dependence. We found that post-fire recovery does not necessarily reverse prior degradation trajectories, consistent with observed changes in canopy structure and composition after severe fires in Australian forests [79]. At the same time, the resilience index suggests that the SMM system exhibits lower structural resilience, driven by slower regeneration rates, climatic constraints, and the cumulative effects of recurrent fires. Similar long-term declines in resilience have been reported in fire-frequent chaparral ecosystems [80]. The shorter time-to-half recovery at MDSF (6 months) compared to the SMM (14 months) further clarifies differences in regeneration strategies between eucalyptus ecosystems and Californian shrublands.

### 4.2. Performance Assessment of Recovery Modeling Approaches

**Figure 9:**
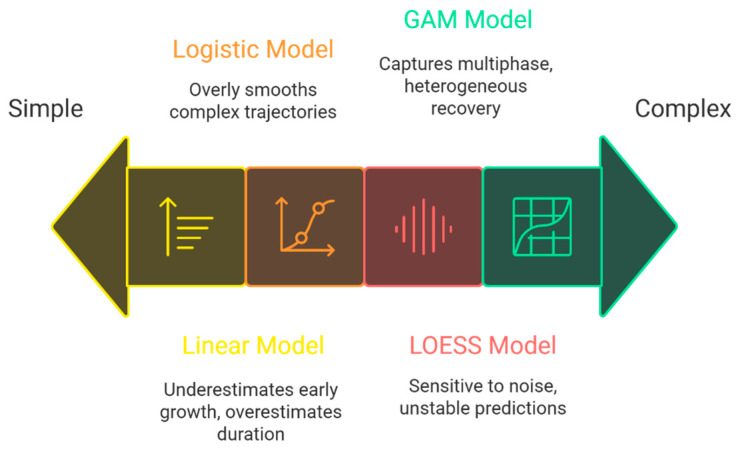
Statistical methods for modeling vegetation recovery after fire.

This study focuses on comparing vegetation recovery models. In both study regions, GAM consistently outperformed other approaches according to Table 5. We found that GAM is well suited to modeling ecological time series (Figure 9) because its flexibility in capturing nonlinear relationships, inflection points, and seasonal fluctuations makes it better able to represent complex, post-disturbance dynamics, in line with [81]. Linear models, although simple and interpretable, performed poorly on most criteria and consistently underestimated early recovery while overestimating the timing of stabilization. The Linear approach, as shown in Table 5, did not yield acceptable results because it assumes that vegetation changes over time occur at a constant slope, whereas post-fire recovery involves an initial rapid-growth phase, a subsequent slowdown, and a final stage characterized by stabilization or secondary oscillations—patterns that clearly deviate from linear behavior [82]. Furthermore, the linear model cannot represent inflection points, threshold effects, or species-specific responses across different fire severities, and thus oversimplifies the recovery trajectory, typically underestimating early growth and overestimating the duration of the stabilization phase. Previous studies have also emphasized that linear models are not suitable for post-disturbance ecological processes, which generally follow nonlinear, time-dependent patterns [83].

In this study, we found that Logistic models often over-smoothed the recovery trajectory or failed to capture late-stage fluctuations, a pattern clearly visible at the SMM, and in contrast to [84], where sigmoidal recovery curves were expected. This may indicate that post-fire trajectories do not follow logistic growth, consistent with results from [85]. Possible reasons include delayed germination, repeated disturbances, or competitive interactions not accounted for in two-parameter models.

By contrast, the LOESS model, unlike the Linear and Logistic approaches, was able to capture a substantial portion of post-fire temporal complexity [83]. We found that, given the structure of LOESS—which imposes no predefined functional form on the data and estimates locally weighted regressions—it can represent short-term changes, small oscillations, and nonlinear vegetation behavior without the need to select an explicit functional form. This is the main reason for LOESS outperforming Linear and Logistic models. This feature allowed LOESS, in both SMM study regions, to more accurately capture early recovery than Logistic and to better represent late stabilization than Linear. Moreover, as seen in Table 5, LOESS produced lower RMSE_cv than GAM in the SMM region. This can be attributed to its data-driven and nonparametric nature (REF). Given the heterogeneous distribution of the data and the fact that LOESS does not require any distributional assumptions (reference), this method, unlike parametric models, does not incorporate the data’s distributional structure into the fitting process and instead relies solely on local smoothing and point-wise weighting. Consequently, under conditions of spatio-temporal heterogeneity, localized fluctuations, or irregular patterns, LOESS may yield lower RMSE_cv—not because it is structurally superior to GAM, but because its local estimates are more sensitive to small-scale variations and, without enforcing a global functional form, it applies more flexible fits to scattered segments of the data. This highlights that the observed differences in RMSE between models are partly due to the uneven spatial and temporal distribution of the data, which affects model sensitivity and error metrics. Therefore, the lower RMSE_cv for LOESS should not be interpreted as evidence of higher accuracy or greater capacity to represent long-term ecological trends, but rather as a reflection of its high sensitivity to local data patterns and the absence of global structural constraints.

Unlike linear or parametric nonlinear models, GAM does not impose a predefined functional form on the recovery trajectory; instead, it estimates the relationship between vegetation index values and time using flexible smoothing functions, typically cubic or thin-plate splines [35,36]. This flexibility is essential for modeling disturbance-driven systems, where recovery is rarely uniform and often involves sudden jumps, periods of stagnation, or secondary oscillations induced by biophysical feedbacks.

In this study, GAM consistently yielded the lowest AIC and BIC values for both the SMM and MDSF datasets, making it the most efficient model in terms of simplicity and performance. In addition, the combination of low in-sample error (RMSE_in) and competitive cross-validation error indicates that the model is not overfitted while still retaining good generalization ability—a balance that is difficult to achieve in ecological time-series modeling. This finding is consistent with earlier work showing that GAM outperforms polynomial and Logistic models in capturing complex successional curves, especially in long-term Landsat time series [59,85,86].

We found that one reason for GAM’s superior performance is its ability to represent multi-phase recovery—a pattern observed in both ecosystems. After severe fires, vegetation typically undergoes a rapid initial growth phase (driven by resprouting or herbaceous expansion), followed by a mid-term slowdown due to competition or climatic stress, and eventually reaches a stabilization phase as structural maturity is approached [78]. Linear and Logistic models cannot adequately represent such multi-phase patterns, whereas GAM reconstructs this behavior without the need to predefine inflection points.

The reason GAM can correctly represent multi-phase recovery lies in the nature of penalized splines, which form the core of the model. Instead of following a closed-form function like Linear or Logistic, GAM’s trend function is constructed from a combination of basic functions, and the curvature of this function is controlled by a smoothing parameter. This mechanism allows the model to capture changes in growth rate at multiple temporal scales so that sudden post-fire increases, competition- or resource-limitation-driven slowdowns, and long-term stabilization phases can each be represented by different segments of the smooth function. Since the smoothing level in GAM is determined in a data-driven way using methods such as GCV or REML, the model introduces inflection points only when the data require them, avoiding artificial structural imposition. This property enables GAM to reconstruct multiple inflection points without explicitly defining breakpoints, because its splines respond independently to structural changes in each part of the time series. In contrast, Linear and Logistic models lack sufficient degrees of freedom to vary curvature over time—the Linear model because of its constant slope, and the Logistic model because it is limited to a single inflection point. Thus, GAM’s ability to represent multi-phase behavior is a direct consequence of its flexible functional architecture and the use of penalties to control curvature.

Compared to other nonlinear methods such as LOESS, our GAM-based framework is better suited for irregular Landsat time series that commonly contain cloud-induced gaps and sensor inconsistencies. GAMs prevent overfitting through automatic smoothness selection via REML/GCV and the use of penalized splines, while also enabling formal statistical inference (e.g., confidence intervals and phase detection). In contrast, LOESS relies on subjective tuning choices and is more prone to local overfitting and boundary effects in uneven data [35,87]. Although LOESS exhibited lower RMSE_cv in some cases, GAM provided greater stability in long-term predictions, which is more important for ecological interpretation. Compared to parametric nonlinear models such as the logistic model, GAM offers greater flexibility by capturing multiple recovery phases without imposing a predefined functional form, thereby avoiding excessive smoothing of late-stage fluctuations [88]. In addition, relative to piecewise approaches such as MARS, GAM produces smoother and more continuous response curves that are better aligned with the gradual nature of vegetation recovery and offer improved ecological interpretability [89].

Unlike machine learning methods such as random forests or neural networks, which often lack transparency and require larger datasets, GAM strikes a suitable balance between flexibility, interpretability, and uncertainty quantification. Recent evidence from post-fire recovery studies further supports the robustness of GAM extensions (e.g., GAMMs) in handling heterogeneity and capturing intensity patterns [90]. Overall, these characteristics position GAM as a reliable and transferable approach for analyzing vegetation recovery using remotely sensed data.

These results demonstrate that the proposed GAM-based framework provides an explicit representation of nonlinear recovery dynamics by jointly capturing (i) the shape of the temporal trajectory, (ii) phase transitions during regrowth, and (iii) stability relative to multiple recovery thresholds (95%, 100%, 105%). Therefore, our approach extends beyond conventional pre-/post-fire comparisons and enables trajectory-based resilience assessments in fire-affected ecosystems.

Although various nonlinear and machine-learning approaches exist for modeling post-disturbance vegetation dynamics, our focus on GAM was intentional due to its interpretability and capability to explicitly reconstruct recovery trajectories. In contrast to tree-based models (e.g., Random Forest) or spline-based regression models such as MARS, which primarily optimize predictive accuracy, GAM provides smooth functions that describe the temporal pathway itself, enabling the detection of multi-phase recovery, inflection points, and stabilization behavior in direct relation to ecological processes. Additionally, while polynomial-based GLMs can represent nonlinearities, they still impose a global functional structure and may introduce artificial curvature. Therefore, GAM offers an effective balance between flexibility and ecological interpretability, making it especially well-suited for trajectory-based resilience assessment in this study.

### 4.3. Limitation and Future Work

Although this study provides a reliable comparative assessment of post-fire recovery using long-term Landsat time series, several limitations should be considered. This analysis is focused on two fire-prone regions that offer meaningful ecological contrasts, but they do not represent the full range of global forest conditions; therefore, the findings should be interpreted within the specific environmental conditions of these landscapes. Another important limitation relates to the quality and temporal stability of the available satellite data, as irregular observation patterns, especially in the SMM site where data availability was highly uneven, made it difficult to capture short-term recovery dynamics. In addition, temporal gaps caused by cloud cover and the Landsat 7 SLC-off issue during 2010–2013 obscured some disturbance signals and limited the ability to conduct more detailed time-series analyses. This study relied on high-resolution imagery for validation, which provided valuable visual reference information, but could not fully replace field measurements, especially in heterogeneous or partially burned areas where conditions vary at small spatial scales. Climate effects were examined using coarse-resolution reanalysis data and simple correlation metrics, which may not fully reflect local, lagged, or nonlinear interactions between climate and vegetation. Furthermore, disturbance detection and recovery assessment were based on a specific set of statistical and change-point models. While this provided a coherent framework for comparison, examining more diverse modeling approaches could reveal additional insights into post-fire ecosystem behavior.

In order to expand and strengthen the findings of the present study, future research may integrate multi-sensor data sources such as Sentinel-2, Landsat Collection 2 ARD, GEDI LiDAR, or combined optical–SAR observations with advanced modeling approaches (e.g., generalized additive models) to improve temporal coverage, reduce data gaps, and consider key structural variables, including canopy height, biomass, and fire severity, inspired by the study of [27] thereby enabling a more comprehensive assessment of post-fire vegetation recovery processes. Establishing field maps or collaborating with environmental monitoring networks would also greatly enhance classification accuracy, burn-severity calibration, and the interpretation of mixed or sub-canopy signals. Future studies would also benefit from incorporating climate and soil variables at higher spatial resolutions and from more explicit analysis of extreme events, lag effects, and combined climate drivers. From a modeling perspective, approaches such as Bayesian hierarchical models, state-space methods, machine-learning sequence models (such as LSTM or temporal convolutional networks) or hybrid physical statistical frameworks could offer a more flexible way to represent complex and nonlinear recovery pathways.

Finally, expanding the analysis to a wider range of fire regimes, vegetation types, and management histories would improve the generalizability of the findings and support the development of more transferable post-fire recovery metrics. Altogether, these efforts increase the potential of remote sensing to capture and understand ecosystem resilience in diverse landscapes.

We acknowledge that more advanced nonlinear and hierarchical frameworks could further enhance the modeling of post-fire recovery dynamics. Approaches such as Random Forest and MARS could provide additional insights into the relative importance of environmental drivers, while Hierarchical GAMs may better capture multi-level ecological structures such as differences among fire severities or vegetation communities. Future studies should incorporate these models to assess model sensitivity and improve predictive performance, while maintaining a clear link between statistical representation and ecological process understanding.

## 5. Conclusions

This study compared long-term Landsat time series in two fire-prone ecosystems—Mediterranean chaparral-oak forests that had repeatedly burned in southern California (USA) and eucalyptus forests affected by the 2019–2020 Black Summer fires in New South Wales (Australia)—to quantify post-fire vegetation recovery and evaluate four widely used modeling approaches. Despite limitations related to data gaps and sensor harmonization, the results revealed completely contrasting recovery trajectories: the Australian site reached 95% of pre-fire levels in about 21 months and returned to baseline within 2 to 5 years, whereas the repeatedly burned U.S. site required 9 to 11 years to approach baseline and never consistently exceeded its historical maximum. Temperature was the main climatic recovery factor in Australia (Spearman ρ = 0.513, *p* < 10^−8^), whereas no climatic variable significantly influenced recovery in California, highlighting the dominant role of fire-adaptive biological traits on short-term climate responses in these systems. Among the tested methods, the GAM clearly outperformed linear regression, logistic growth, and LOESS across all metrics (AIC, BIC, RMSE_in, RMSE_cv) because GAM’s flexibility allowed it to accurately capture the typical multi-phase recovery pattern—rapid initial regrowth, slowed mid-phase growth, and stabilization in later stages—while linear models underestimated early recovery and overestimated total duration, logistic models overly smoothed complex trajectories, and LOESS was overly sensitive to noise and produced unstable long-term predictions. As a result, these findings indicate that simple parametric models are insufficient to represent actual post-fire recovery dynamics, and only flexible semi-parametric approaches such as GAM provide reliable and ecologically meaningful estimates of recovery time and resilience. Although it was limited to two ecosystems, uneven coverage before 2013, coarse climate data, and four modeling approaches, this study established a robust and transferable workflow by combining PELT change-point detection, slope analysis, and GAM-based smoothing. Future research should incorporate high-frequency and multi-sensor data, finer-scale and lagged climate analyses, field validation, and advanced sequence models (such as Bayesian state-space models and LSTM/Transformer) to further refine recovery predictions under intensifying fire regimes. Ultimately, this work confirms GAM as the current gold-standard statistical tool for satellite-based post-fire recovery assessment and emphasizes that embracing methodological complexity—rather than assuming linear or sigmoidal forms—is essential for accurate estimation of ecosystem resilience and for informing adaptive fire management in a warming world.

## Figures and Tables

**Table 1:** Summary of the remote sensing and climate datasets used in this study, including data reliability, temporal and spatial resolution, number of observations, study period, and data sources for MDSF and SMM.

Data Source	Year		Number of Data		Spatial Resolution	Temporal Resolution	Data Reliability
Region	SMM	MDSF	SMM	MDSF			
Landsat 5	2007–2010	-	37	-	30 m (multispectral), 120 m (thermal)	16 days	RMSE < 12 m; Radiometric accuracy: 5%
Landsat 8	2013–2024	2015–2024	88	110	30 m (multispectral), 100 m (thermal), 15 m (panchromatic)		RMSE < 12 m; Radiometric accuracy: 3%
Precipitation	2007–2024	2015–2024	125	110	0.25° (~31 km)	Monthly (aggregated from hourly ERA5 data)	High (reanalysis using satellite + ground observations)
Temperature						Monthly (mean of hourly ERA5 data)	High (validated atmospheric reanalysis)
Soil Moisture							Moderate–High (satellite assimilation + land model)

**Table 2:** Overview of the statistical and ecological metrics used to evaluate recovery models. While statistical measures such as AIC, BIC, and RMSE describe how well each model fits the data and how reliably it predicts vegetation trends, the ecological indicators capture how the ecosystem itself responds how fast it recovers, how resilient it is, and how far it deviates from its pre-fire condition. Together, these metrics provide a balanced and meaningful picture of both model performance and real ecological behavior.

Metric	Type	Formula/Definition	Interpretation and Application
AIC (Akaike Information Criterion)	Evaluation	$n\;ln(\frac{RSS}{n})+2k$	Measures the trade-off between model fit and complexity. Lower AIC indicates a more efficient model with better parsimony [61,62].
BIC (Bayesian Information Criterion)		$n\;ln(\frac{RSS}{n})+kln(n)$	Similarly to AIC but penalizes model complexity more strongly. Lower BIC reflects preference for simpler, more generalizable models [63].
RMSE-in (In-sample Root Mean Square Error)		$\sqrt{(1/n){\Sigma (y\_i-ŷ\_i)}^{2}}$	Quantifies model fit on the training data. Smaller values indicate better in-sample accuracy, though not necessarily better generalization [64].
RMSE-CV (Cross-validation RMSE)		((1/N)ΣΣ(y_i−ŷ_i^(−j))2)	Evaluates model performance on unseen data using cross-validation. Lower RMSE-CV reflects higher predictive stability and reduced overfitting [65].
Recovery_rate	Ecological	$\frac{\left(y_{target}-y_{min}\right)}{\left(y_{pre}-y_{min}\right)}\times \frac{1}{\left(t\_rec-t\_min\right)}$	Ecological metric quantifying vegetation recovery speed. Higher values indicate faster ecosystem regeneration after fire disturbance [66].
Time to half months		$\frac{T}{2}=t\;when\frac{\left(y\_t-y\_min\right)}{\left(y\_max-y\_min\right)}=0.5$	Time required for vegetation to recover to 50% of pre-disturbance state. Shorter values indicate faster ecological recovery.
Resilience index		$\frac{\left(y\_post-y\_min\right)}{\left(y\_pre-y\_min\right)}$	Measures the ecosystem’s ability to return to pre-disturbance conditions. Higher RI indicates stronger resilience [67]
Hysteresis index		$\frac{\int |y\_rec(t)-y\_dec(t)|dt}{\int y\_dec(t)dt}$	Quantifies asymmetry between degradation and recovery pathways. Higher HI reflects path-dependence and ecological instability [68].
Nadir Date		argmin(y_t)	The date when vegetation reaches its minimum level post-disturbance, marking the true impact point and start of recovery.

**Table 3:** Correlation statistics between climatic variables (temperature, precipitation, and soil moisture) and vegetation dynamics derived from the EVI index for the SMM and MDSF. Pearson (r) and Spearman (ρ) coefficients, along with their *p*-values, are reported for each region.

Variable	Region	Pearson_r	Pearson_p	Spearman_r	Spearman_p
Temperature	SMM	−0.045	0.620	−0.088	0.331
	MDSF	0.155	0.105	0.513	1.14 × 10^−8^
Precipitation	SMM	−0.003	0.972	0.026	0.769
	MDSF	−0.076	0.428	0.105	0.273
Soil moisture	SMM	0.074	0.415	0.136	0.136
	MDSF	−0.034	0.723	−0.150	0.118

**Table 4:** The recovery dynamics estimated by four modelling approaches—Linear, LOESS, Logistic, and GAM—across the SMM and MDSF regions are summarised in this table. Recovery rate, time to half recovery, resilience index, hysteresis index, and estimated nadir dates are among the reported indicators that show significant variations in how each model depicts the severity, duration, and speed of disruptions. These differences show how the models’ sensitivity to structural breaks and nonlinear rebound patterns varies by region.

Region	Model	Recovery Rate	Time to Half Months	Resilience Index	Hysteresis Index	Nadir Date
SMM	Linear	2.143	14	0.712	0.147	15 February 2019
	LOESS	4.988		1.224	0.117	
	Logistic	1.76		0.369	0.113	
	GAM	4.760		1.112	0.117	
MDSF	Linear	2.143	6	0.983	0.548	15 April 2020
	LOESS	1.229		1.013	0.547	
	Logistic	0.964		1.006	0.548	
	GAM	1.890		1.004	0.542	

**Table 5:** Summary of model evaluation metrics including AIC, BIC, RMSE_in, and RMSE_cv for four statistical modeling approaches (Linear, LOESS, Logistic, and GAM) applied to SMM and MDSF datasets.

Method	Region	AIC	BIC	RMSE_in	RMSE_cv
Linear	SMM	162.967	165.091	45.299	117.004
	MDSF	103.739	109.091	0.734	2.069
LOESS	SMM	147.246	150.148	24.927	109.380
	MDSF	91.289	97.9	0.627	2.384
Logistic	SMM	168.256	171.962	47.2	113.362
	MDSF	104.162	111.299	0.721	2.380
GAM	SMM	142.887	146.377	20.396	112.862
	MDSF	46.699	64.395	0.328	2.260

**Figure A1:**
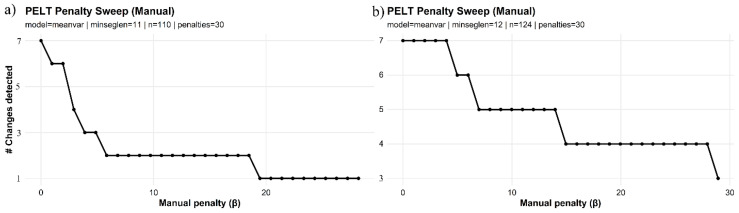
Comparison of the number of detected changepoints in the PELT method with increasing penalty (β) for region (**a**) MDSF and (**b**) SMM. The plots show that as β increases, the sensitivity of the algorithm decreases and fewer changepoints are detected.

**Figure A2:**
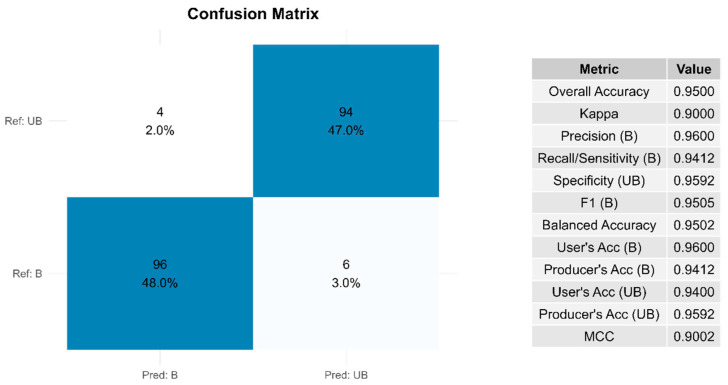
Confusion matrix and classification performance metrics for the MDSF. The matrix summarizes the agreement between predicted and reference classes (B and UB), with the diagonal cells representing correctly classified samples. The accompanying table reports key accuracy indices, including overall accuracy, Cohen’s kappa, precision, recall, specificity, F1-score, balanced accuracy, user’s and producer’s accuracies for both classes, and the Matthews correlation coefficient (MCC). These metrics collectively indicate strong classifier performance and high reliability in distinguishing between the two classes.

**Figure A3:**
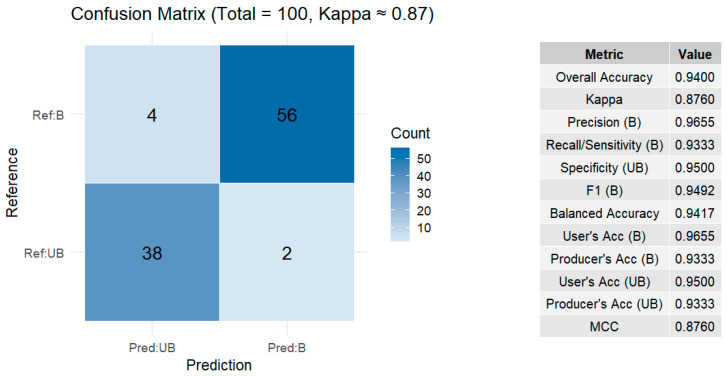
Confusion matrix and accuracy metrics for the Support Vector Machine (SVM) classification of burned areas in the SMM region using Landsat surface reflectance data in Google Earth Engine. The matrix is based on 100 stratified random validation points. Class “B” denotes burned pixels and “UB” denotes unburned pixels. Overall accuracy = 94.00%, Kappa coefficient ≈ 0.87, Producer’s accuracy (burned) = 93.33%, User’s accuracy (burned) = 96.55%.

**Figure A4:**
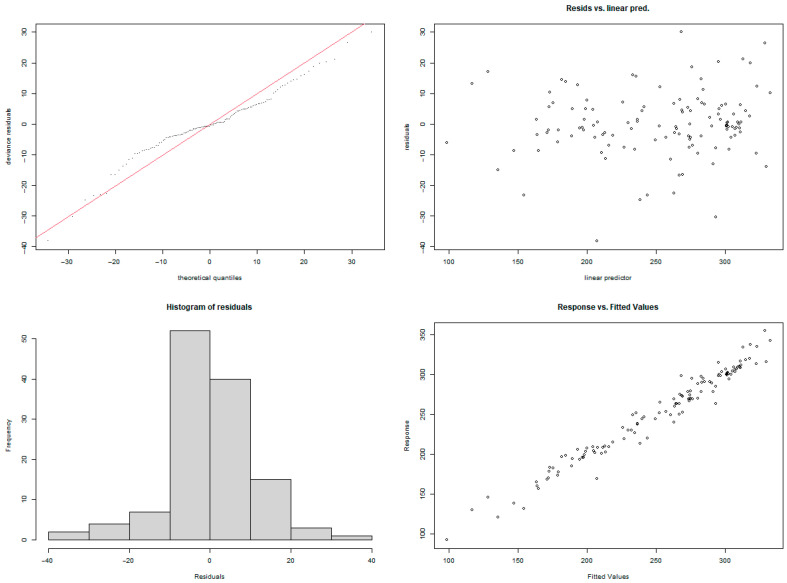
Diagnostic plots of the linear regression model for the SMM region, including the QQ plot, the residuals versus fitted values plot, the histogram of residuals, and the plot of observed versus fitted values. These plots were used to evaluate the model assumptions and the quality of fit. Based on these diagnostics, a value of k = 65 was selected.

**Figure A5:**
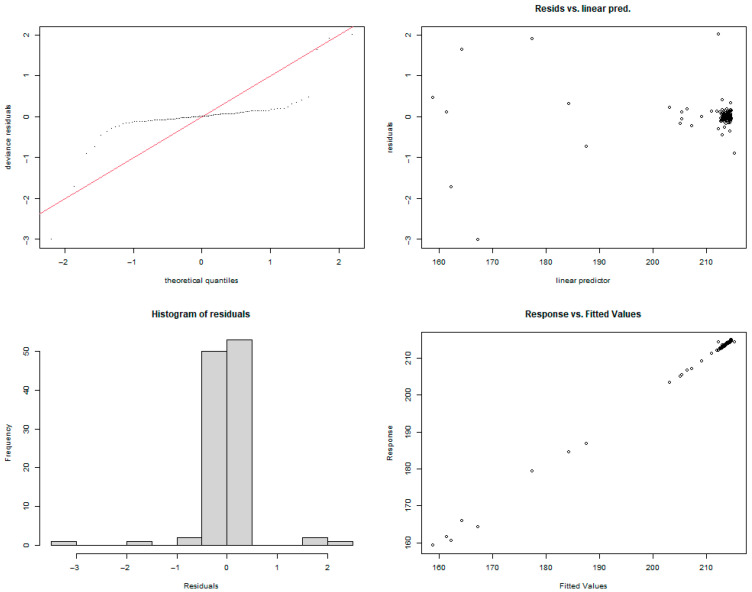
Diagnostic plots of the linear regression model for the MDSF region, including the QQ plot, residuals versus linear predictor, histogram of residuals, and the response versus fitted values. These plots were used to assess model assumptions and goodness of fit. Based on these diagnostics, a value of k = 45 was selected.

**Figure A6:**
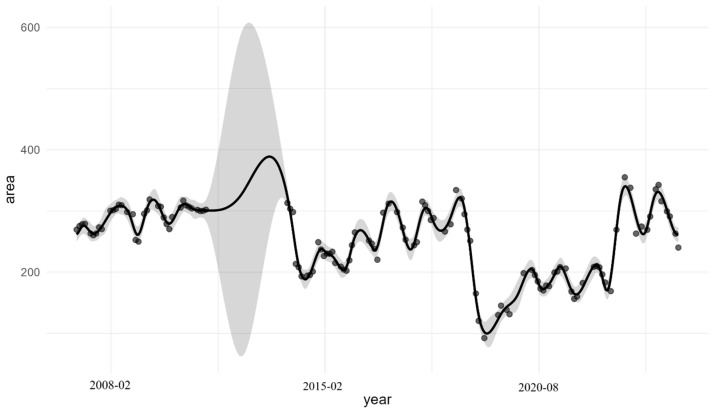
Smoothed time-series profile for the United States region using a moving window of k=45. The shaded band represents the estimated uncertainty. The substantial widening of the uncertainty interval around $1.3\times 10^{9}$ reflects a data-gap in this segment, causing reduced estimation reliability compared to adjacent regions.

**Figure A7:**
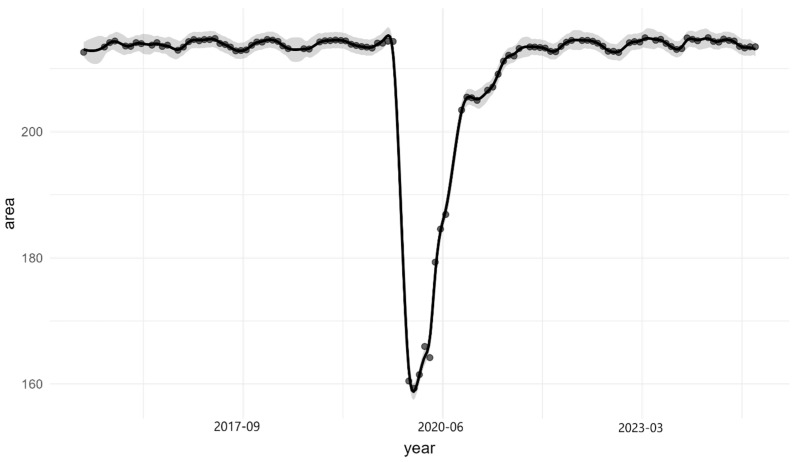
Smoothed time-series profile of the MDSF region obtained using a moving window size of k=45. The plot illustrates the central trend along with the associated confidence band, highlighting both the stable behavior of the signal and the pronounced drop observed around $1.6\times 10^{9}$ on the time axis.

**Figure A8:**
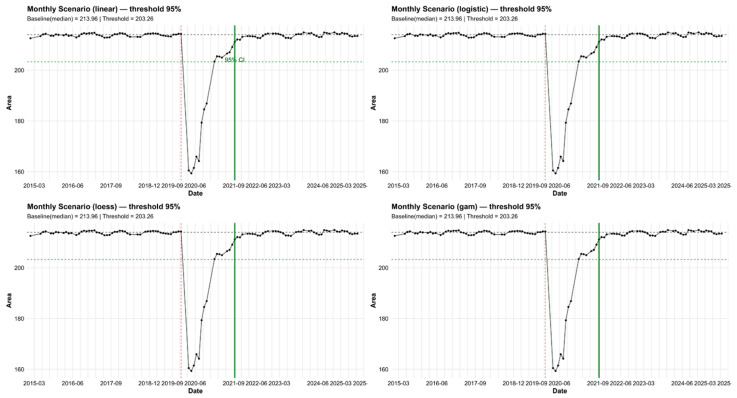
Monthly area dynamics in the MDSF, modeled using four approaches (linear, logistic, loess, and GAM) with a 95% threshold for anomaly detection. The dashed horizontal line indicates the baseline median (213.96), and the green dashed line represents the 95% threshold (203.26). The vertical red dashed line marks the onset of the disturbance event, while the green solid line denotes the model-based recovery date. All modelling scenarios show a pronounced decline in area during early 2020 followed by a gradual return toward baseline conditions, with minor variation in recovery timing among models.

**Figure A9:**
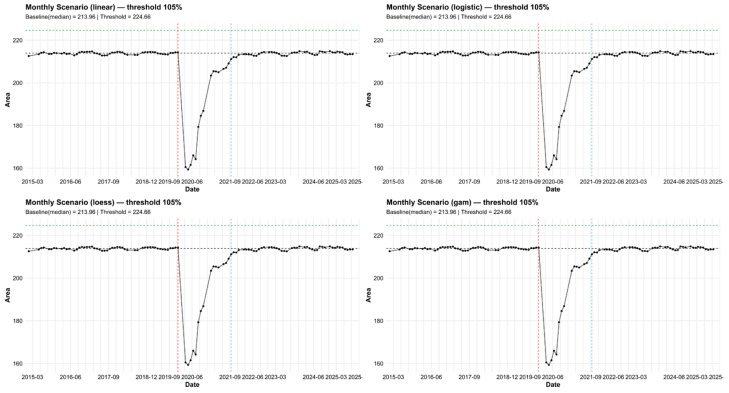
Monthly area trajectories for the MDSF under four modelling approaches—linear, logistic, loess, and GAM—using a 105% recovery threshold. The dashed horizontal line represents the baseline median (213.96), while the green dashed line marks the 105% threshold (224.66). The red vertical dashed line indicates the disturbance onset in early 2020, followed by a sharp decline in area. The blue vertical dashed line denotes the model-specific recovery date at which the post-disturbance trajectory surpasses the 105% recovery threshold. All models capture a rapid post-disturbance increase, with minor variation in estimated recovery timing across modelling frameworks.

**Figure A10:**
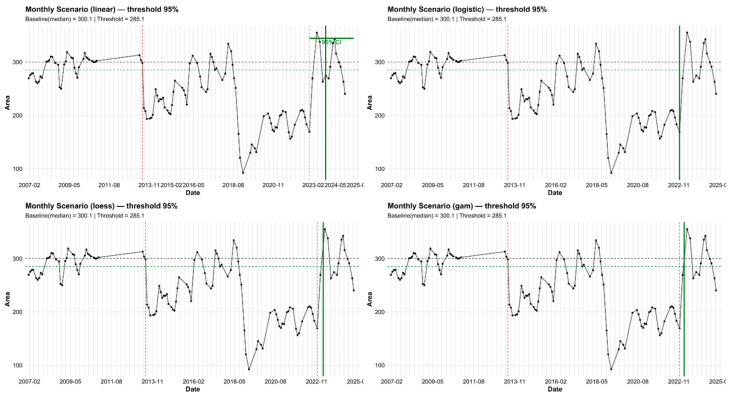
Monthly anomaly detection results for the SMM under four modeling approaches (linear, logistic, loess, and GAM) using a 95% threshold criterion. The black points represent observed monthly area values, and the dashed horizontal lines indicate the baseline median (300.1) and the anomaly detection threshold (285.1). The vertical red dashed line denotes the onset of the disturbance period, while the green vertical lines mark the first detected anomalous month for each model. All four methods consistently identify substantial declines during the disturbance period, highlighting the robustness of the anomaly-detection framework for SMM study region.

**Figure A11:**
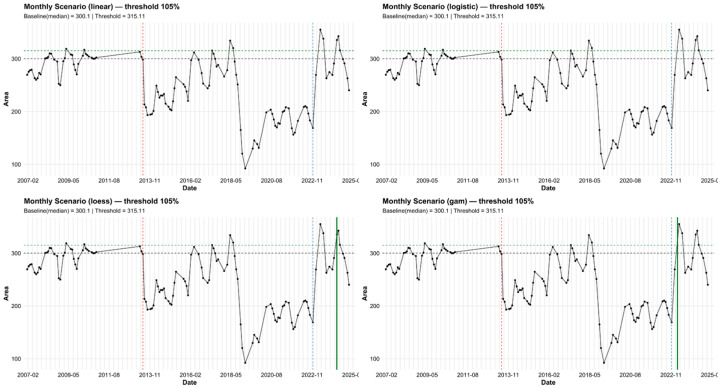
Monthly anomaly detection results for the SMM using four modeling approaches (linear, logistic, loess, and GAM) with a 105% exceedance threshold. Black points represent observed monthly area values, and gray lines show temporal variability. The horizontal dashed green line indicates the baseline median (300.1), while the red dashed vertical line marks the estimated onset of deviation from baseline conditions. The blue and green vertical lines denote model-specific exceedance points relative to the 315.11 threshold. All models consistently identify anomalous increases in area during 2022–2024.

## Data Availability

The required data and codes are available at https://zenodo.org/records/17807463 (accessed on 1 October 2025). Furthermore, The GEE code used preprocessing image collections is available at https://zenodo.org/records/17807463 (accessed on 1 October 2025).

## Appendix A

Refer to https://zenodo.org/records/17807463 (accessed on 1 October 2025).

## Appendix B

**Table A1:** Comparison of model performance metrics across different recovery scenarios for the USA and AUS regions. The table reports RMSE, R^2^, and MAE values for span levels from 0.1 to 0.5 under recovery factors of 0.95, 1, and 1.05, providing a detailed assessment of model sensitivity and stability.

Region		USA Recovery			AUS Recovery		
Span	Evaluation Criteria	0.95	1	1.05	0.95	1	1.05
0.1	RMSE	24.3	25.9	27.1	25.4	27.6	26.1
	R^2^	0.91	0.86	0.85	0.87	0.84	0.85
	MAE	17.4	19.9	20.7	19.1	21.8	20.5
0.2	RMSE	23.4	25.1	25.8	24.5	25.9	25.1
	R^2^	0.92	0.88	0.87	0.88	0.86	0.87
	MAE	17.6	19.4	19.9	18.5	20.1	19.6
0.3	RMSE	23.1	24.3	24.7	23.9	24.6	24.0
	R^2^	0.92	0.89	0.88	0.89	0.87	0.88
	MAE	17.8	18.9	19.3	18.1	19.2	19.0
0.4	RMSE	24.8	23.1	23.4	22.0	23.1	22.4
	R^2^	0.91	0.93	0.94	0.92	0.93	0.92
	MAE	19.1	17.4	17.8	17.1	17.9	17.5
0.5	RMSE	27.0	24.9	25.4	24.2	25.6	25.3
	R^2^	0.89	0.9	0.89	0.88	0.88	0.89
	MAE	21.1	18.9	19.5	18.4	20.0	19.8
